# Efficacy and safety of parathyroid perfusion assessment by fine-needle prick during thyroid surgery: a prospective study

**DOI:** 10.3389/fendo.2025.1498083

**Published:** 2025-02-27

**Authors:** Peisong Wang, Haowen Xue, Xin Wang, Shuai Xue

**Affiliations:** General Surgery Center, Department of Thyroid Surgery, The 1st Hospital of Jilin University, Changchun, Jilin, China

**Keywords:** needle prick, ICG, thyroid surgery, prospective study, parathyroid

## Abstract

**Objective:**

This prospective study aimed to analyze the efficacy and safety of fine-needle prick (FNP) in the assessment of parathyroid gland (PG) perfusion during thyroid surgery.

**Methods:**

A total of 147 patients with papillary thyroid carcinoma (PTC) who underwent lobectomy with therapeutic unilateral central lymph node dissection (CLND) performed by the same surgeon at the First Hospital of Jilin University between June and September 2024 were included in this prospective study. Following removal of the thyroid and unilateral central lymph nodes, indocyanine green (ICG) was prepared and administered intravenously. Fluorescence signals and images were captured using a near-infrared system to obtain the ICG1 score. Approximately 5–10 min thereafter, the fluorescent signal dissipated, after which the preserved PG was evaluated using FNP. An FNP score was established based on oozing blood from the parathyroid. After hemostasis was achieved by compression with gauze, ICG was reinjected to reassess PG perfusion, which yields the ICG2 score.

**Results:**

In 13 patients, only one parathyroid was identified, while the other was inadvertently excised during surgery. A total of 269 (95.73%) parathyroids were evaluated, with consistent scores observed between the use of the ICG1 and FNP methods. However, in six congested PGs with a darkened appearance, the FNP method could not accurately assess blood supply due to the slow oozing of accumulated blood following the procedure. The Spearman’s correlation coefficient between the ICG1 and FNP methods was 0.973 (*p* < 0.001), demonstrating strong concordance in determining PG blood supply. Furthermore, the identical ICG1 and ICG2 scores indicated that FNP did not adversely affect PG perfusion.

**Conclusion:**

The FNP test could represent a safer, simpler, and more reliable alternative to ICG for the assessment of PG perfusion. However, further validation in patients undergoing total thyroidectomy and bilateral CLND is warranted.

**Clinical trial registration:**

https://www.chictr.org.cn/, identifier ChiCTR2400084531.

## Introduction

The incidence of thyroid cancer has increased dramatically worldwide, with the rapid rise in differentiated thyroid carcinoma (DTC) being the primary contributor to this escalating trend (1). Surgery remains the most common treatment modality for DTC (2). With the increasing frequency of thyroidectomy, particularly total thyroidectomy (TT), postoperative permanent hypoparathyroidism has emerged as the most severe complication for patients with DTC (3). Studies have indicated that permanent hypoparathyroidism can significantly reduce the quality of life and increase the patient mortality rates (4). Consequently, ensuring parathyroid protection during thyroidectomy is a critical concern for surgeons.

Parathyroid location and perfusion are key determinants in their preservation during thyroidectomy. Various methods, including nanoparticles, laser speckle contrast imaging, and near-infrared autofluorescence (NIAF), have been employed intraoperatively to identify the parathyroid glands (PGs), which have shown promising outcomes (5–8). However, safeguarding the delicate vessels that ensure parathyroid perfusion remains challenging. Accurate assessment of parathyroid perfusion is essential in order to determine whether to autotransplant the gland or to preserve it *in situ*. Indocyanine green (ICG) is frequently utilized to evaluate parathyroid perfusion (9). Patients undergoing TT with one or more well-vascularized PGs typically exhibit higher serum calcium and parathyroid hormone (PTH) levels (10). However, the detection of ICG fluorescence requires costly equipment, and some patients may experience allergic reactions to ICG. Its use during surgery also increases both the cost for patients and the time required for surgeons (11). Thus, there is a need to develop a more convenient and feasible method for the evaluation of the parathyroid blood supply during surgery.

Recently, fine-needle prick (FNP) tests have been utilized to predict parathyroid function (12). Patients with PGs that exhibit excellent vascularity, as assessed using FNP, demonstrate normal PTH levels (12). However, this finding has not yet been validated in other cohorts. To prevent hypoparathyroidism, patients undergoing hemithyroidectomy with assessment of two parathyroids are chosen as an initial step to documenting the efficacy and safety of the technique. Whether the FNP test offers the same efficacy and safety as ICG for parathyroid perfusion assessment remains unclear. Therefore, this prospective study aimed to analyze the efficacy and safety of using FNP to assess parathyroid perfusion during thyroid surgery.

## Materials and methods

### Patients

**Figure 1:**
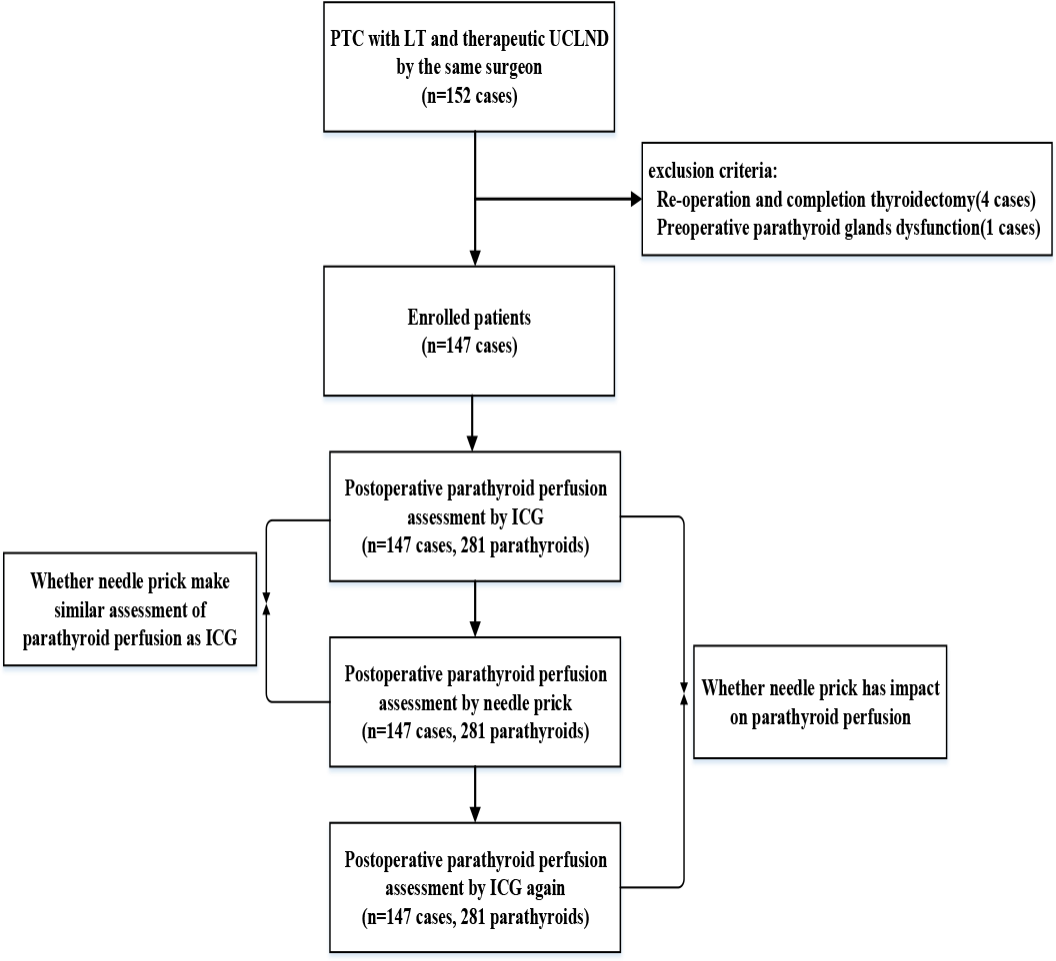
Flowchart of the study design and patient selection.

The inclusion criteria were: 1) papillary thyroid carcinoma (PTC) or central metastatic lymph nodes diagnosed preoperatively by fine-needle aspiration (FNA); 2) lobectomy with therapeutic unilateral central lymph node dissection (CLND); 3) pathologically confirmed PTC and central lymph node metastasis; and 4) surgery performed by the same surgeon. The exclusion criteria were: 1) reoperation and completion of thyroidectomy; 2) age <18 years; and 3) preoperative PG dysfunction. Based on previous therapeutic efficacy and general statistical requirements, the calculated sample size was 152 cases, with the equivalent threshold value set at 0.15 and the average effective rate at 0.90. A total of 147 cases with PTC who underwent lobectomy with therapeutic unilateral CLND performed by the same surgeon (PS Wang) at the present hospital between June and September 2024 were included in this prospective study, as shown in **Figure 1**. This research was approved by the Ethics Committee of the present hospital, and informed consent was obtained from all participants included in the prospective study. The study was registered in the Chinese Clinical Trial Registry (https://www.chictr.org.cn/; registration no. ChiCTR2400084531).

### Surgical procedures

**Figure 2:**
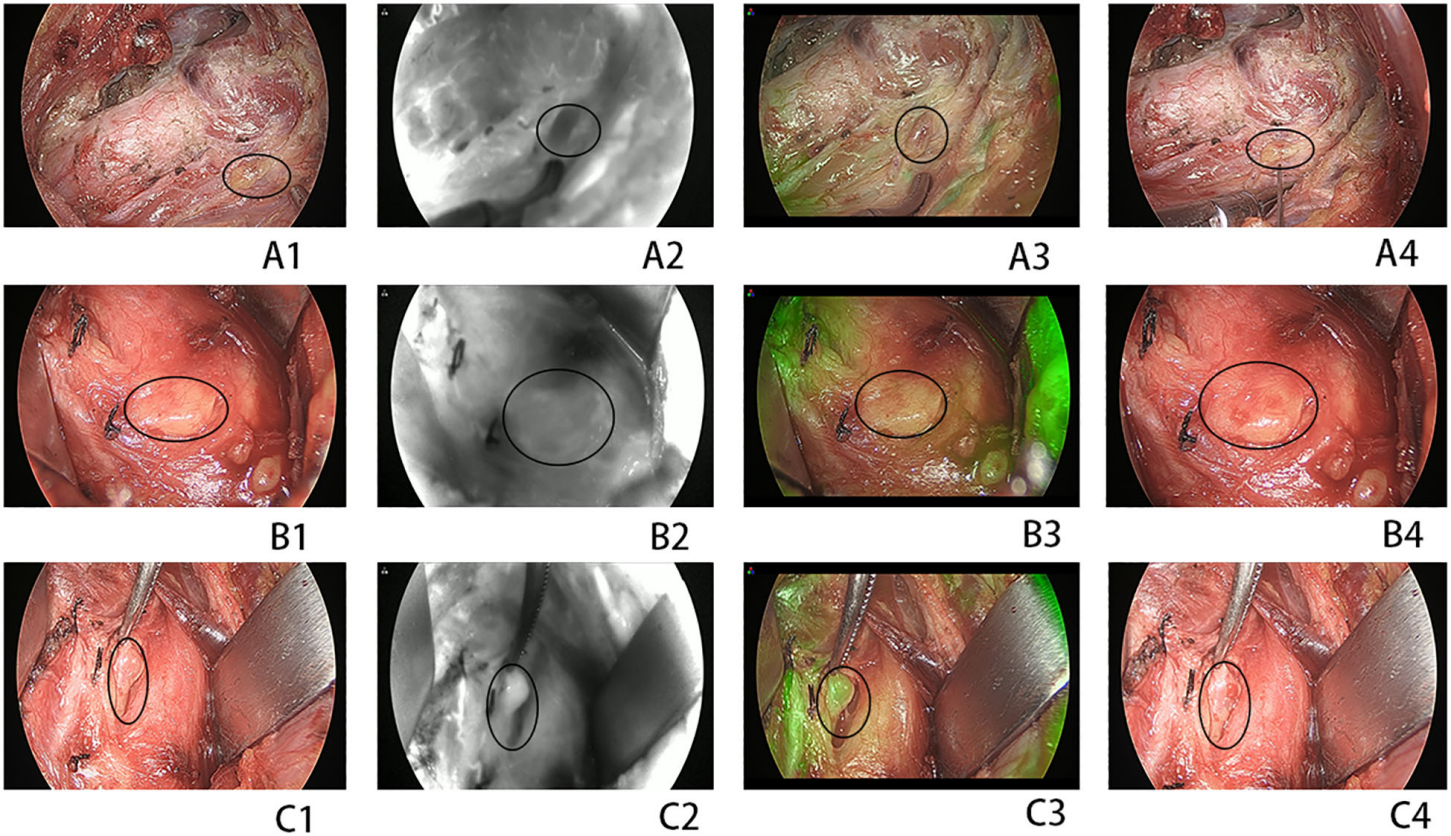
**(A1–A4)** A devascularized parathyroid gland (PG) [indocyanine green (ICG) and fine-needle prick (FNP) score of 0]. **(B1–B4)** A moderately well-vascularized PG (ICG and FNP of score 1). **(C1–C4)** A well-vascularized FNP (ICG and FNP of score 2). **(A1, C1)** Normal view. **(A2, C2)** Near-infrared view. **(A3, C3)** Combined normal and near-infrared view. **(A4, C4)** Normal view after FNP. *Circles* indicate the PG.

FNA confirmation is recommended for suspected thyroid and lymph node nodules. Carbon nanoparticles (CNs) were used to identify parathyroid tissue. CN injections and operation were conducted according to a method described previously (5). During the surgical procedure, the thyroid capsule was meticulously dissected to protect the PG. After removal of the thyroid and unilateral central lymph nodes, ICG was prepared and administered intravenously. Fluorescence signals and images were captured using a near-infrared system to obtain the ICG1 score. Each identified PG was evaluated using ICG fluorescence and classified with ICG scores of 0, 1, and 2, as previously reported (10). Approximately 5–10 min thereafter, the fluorescent signal dissipated. The preserved PG was then examined using FNP. The preserved PG was pricked with a 25-G injection needle. If no blood oozed from the puncture site after the first prick, a second prick was performed. Up to three punctures were allowed. The needle was inserted into the PG parenchyma to a depth of 3–5 mm from the transverse axis of the PG, ensuring that the needle tip remained clear of the PG hilus. An FNP score was established based on the oozing of blood from the parathyroid as follows: FNP0, no blood oozing; FNP1, a small amount of blood oozing slowly and persisting after wiping with gauze; and FNP2, blood effusing rapidly and persisting after clearing. After hemostasis was achieved by compression with gauze, ICG was reinjected to assess PG perfusion, which gives the ICG2 score. The lowest scores for ICG and FNP were used in cases of discordance during the assessment. Representative ICG and FNP images of the PG are shown in **Figure 2**. If an accidentally revascularized PG was identified, parathyroid autotransplantation (PA) into the sternocleidomastoid muscle pocket was performed immediately after confirmation by the frozen section. PA was conducted in accordance with the American Thyroid Association guidelines for postoperative hypoparathyroidism (13).

### Data collection

The following data were collected: age, sex, body mass index (BMI), largest tumor diameter (LTD), tumor number, primary tumor T stage, and metastatic central lymph nodes (MCLNs). The ICG and FNP scores were also recorded during surgery.

### Statistical analysis

The SPSS 26 software was used to statistically analyze the data. Categorical data were described as absolute numbers and were compared using the chi-squared or *F* test. Continuous data with normal and non-normal distributions were expressed as mean ± SD and median with range, respectively, and were analyzed using the *t*-test or *U*-test. Spearman’s coefficients were calculated to assess the consistency of the ICG and FNP scores, and the significance level was set at 0.05.

## Results

### Clinicopathological features of cases with lobectomy and UCLND

Between June and September 2024, 147 consecutive patients underwent lobectomy with therapeutic unilateral CLND performed by the same surgeon (Dr. Wang). A flowchart is shown in **Figure 1**. There were 134 patients who had two PGs and 13 patients who had only one PG identified during the surgical procedure. **Table 1** presents the clinical data of the 147 cases who underwent lobectomy with therapeutic unilateral CLND, followed by parathyroid gland ICG and FNP tests. As shown in **Table 1**, 51 male and 96 female patients were included, with an average age of 42.4 years. In the 147 patients, the median tumor diameter was 1.2 cm, and the tumor number was 3. There were 48 patients diagnosed with Hashimoto’s thyroiditis. The median number of MCLNs was 4, ranging from 2 to 21, while the median number of removed lymph nodes was 11, ranging from 5 to 30. In the 13 patients, only one parathyroid was identified, with the other parathyroid unintentionally resected during surgery. A total of 104 parathyroids without ICG1 or ICG2 signals were autotransplanted into the sternocleidomastoid muscle.

**Table 1:** Clinicopathologic characteristics of all enrolled patients.

Variables	Patients (n=147)
Age, years (mean ± SD)	42.4±11.6
Gender	
Male (%) Female (%)	51 (34.6) 96 (65.4)
BMI (median [range])	25.1 [15.8-37.9]
HT	
Yes (%) No (%)	48 (32.7) 99 (67.3)
LTD,cm (median [range])	1.2 [0.2-5.5]
Tumor number (median [range])	3 [1-8]
T stage	
T1 (%) T2 (%) T3 (%) T4 (%)	84 (57.1) 26 (17.7) 37 (25.2) 0 (0)
CLND	
MCLN (median [range]) RCLN (median [range])	4 [2-21] 11 [5-30]
Parathyroid gland	
Identified number (median [range]) Resected number (median [range]) Implanted number (median [range])	2 [1-2] 0 [0-1] 0 [0-2]

SD, standard deviation; BMI, body mass index; HT, Hashimoto’s thyroiditis; LTD, largest tumor diameter; cN1, clinical metastatic lymph node; CLND, central lymph node dissection; MCLM, metastatic central lymph node; RCLN, removed central lymph node; LLNM, lateral lymph node metastasis; RAI, radioactive iodine ablation.

### Consistency of the FNP and ICG scores for assessing parathyroid perfusion

**Figure 3:**
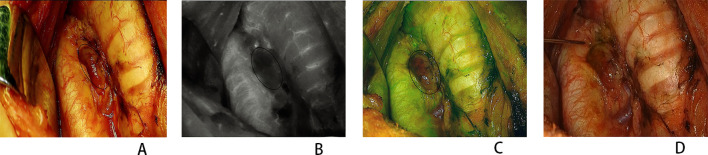
Congested parathyroid gland (PG) perfusion assessment. **(A)** Normal view. **(B)** Near-infrared view. **(C)** Combined normal and near-infrared view. **(D)** Normal view after fine-needle prick (FNP). *Circles* indicate the PG.

**Table 2** shows the 281 parathyroids evaluated, with the same scores obtained using the ICG1 and FNP methods in 269 (95.73%) parathyroids. Both methods determined the presence of blood supply to the PG, but differed in their assessment of the degree of blood supply in six parathyroids. In another six congested PGs with a dark color, the FNP method could misjudge the blood supply of the PG due to the accumulated blood in the parathyroid slowly oozing out after the procedure (**Figure 3**). Comparison of the ICG1 and FNP methods gave a Spearman’s correlation coefficient of 0.973, with *p*-values less than 0.001. There was high consistency between the two methods in determining parathyroid blood supply. Moreover, the ICG1 and ICG2 scores were identical, indicating that FNP did not influence parathyroid perfusion.

**Table 2:** Parathyroid scores assessed by ICG and needle prick.

	Needle prick*			ICG2		
	0	1	2	0	1	2
ICG1						
0	98	6	0	104	0	0
1	0	52	3	0	55	0
2	0	3	119	0	0	122

*Kendall's tau-b and Spearman correlation coefficients are 0.961and 0.973 respectively with both *P* value less than 0.001.

ICG1, postoperative indocyanine green fluorescence imaging for the first time.

ICG2, postoperative indocyanine green fluorescence imaging for the second time after needle prick.

## Discussion

A precise evaluation of parathyroid perfusion during thyroid surgery is crucial as it determines whether to preserve the parathyroid *in situ* or to perform autotransplantation (13). Recent studies have increasingly demonstrated that the ICG score, based on intraoperative ICG angiography, can predict PG function (9, 11, 14–17). This score is a valuable tool for the prediction of hypoparathyroidism following TT (14, 17). A single parathyroid gland ICG score of 2 accurately predicted postoperative normocalcemia during TT (10). However, the unavailability of equipment remains a major limitation when performing ICG during thyroidectomy or parathyroidectomy (11). The use of ICG during thyroid and parathyroid surgical procedures increases the time and overall cost of surgery (11). FNP has been reported as an easy and a time-saving method for predicting parathyroid function; however, whether FNP matches the diagnostic accuracy of ICG in assessing parathyroid perfusion remains unknown.

According to the results, the diagnostic consistency rate of the FNP and ICG methods was as high as 95.73%, indicating that FNP can efficiently predict postoperative parathyroid function. An article on the prediction of parathyroid blood supply using ICG scores concluded that postoperative patients with a well-vascularized PG and an ICG score of 2 do not experience parathyroid dysfunction (10). Wang et al. also found normal postoperative PTH levels in 25 cases with only one well-vascularized PG, as assessed by the FNP test (12). These two methods rule out the possibility of cases developing hypoparathyroidism, which further suggests a high degree of consistency between FNP and ICG in predicting parathyroid function. Moreover, many surgeons are concerned about whether the FNP method injures the parathyroid blood vessels and influences perfusion. The ICG1 and ICG2 scores in this study were identical, indicating that FNP did not negatively impact parathyroid perfusion. The FNP method is simple and easy to use and does not increase the surgical time or cost.

Both methods determined the presence of blood supply to the PG, but differed in their assessment of the degree of blood supply in six parathyroids. This difference does not change the fact that the six PGs were preserved *in situ*. Another six congested PGs with a dark color were easily misjudged when using the FNP method due to the accumulated blood in the parathyroid slowly oozing out after the procedure. In this situation, we waited a few seconds until the remaining blood in the PG was completely discharged. Subsequently, we checked whether fresh blood flowed out of the PG to accurately determine its perfusion.

The FNP test makes the precise identification of PG difficult. Even the most experienced thyroid surgeons can misinterpret other anatomical structures, such as lymph nodes and PGs. If the FNP test demonstrated good vascularization, it could cause false reassurance. In this study, CNs were routinely used for parathyroid tissue identification. An immunochromatographic test strip to detect the PTH for parathyroid identification may be a promising method to assist surgeons in identifying PGs (18).

There are some limitations in this study. Firstly, the PGs identified during surgery were not verified with PTH measurement in their tissue. A very small amount of fat tissue may have been mistakenly recognized as PG, which could have been mixed in with the research sample. Secondly, several risk factors were not investigated, such as location and specific parathyroid conditions (superior or inferior PG). Finally, only those patients who underwent lobectomy and therapeutic unilateral CLND were enrolled, owing to PG safety considerations, which made it impossible to analyze postoperative hypoparathyroidism and the hypocalcemia levels. In the future, we will include patients who have undergone TT and bilateral CLND for a more comprehensive evaluation.

## Conclusion

The FNP test could be a safer, simpler, and more reliable alternative to ICG for the assessment of PG perfusion. However, this finding requires further validation in patients undergoing TT and bilateral CLND.

## Data Availability

The raw data supporting the conclusions of this article will be made available by the authors, without undue reservation.
